# Qige Huxin Formula Attenuates Isoprenaline-Induced Cardiac Fibrosis in Mice via Modulating Gut Microbiota and Protecting Intestinal Integrity

**DOI:** 10.1155/2022/2894659

**Published:** 2022-07-20

**Authors:** Lipeng Shi, Xuqin Du, Biao Zuo, Jinyuan Hu, Wenfu Cao

**Affiliations:** ^1^College of Traditional Chinese Medicine, Chongqing Medical University, Chongqing 400016, China; ^2^Chongqing Key Laboratory of Traditional Chinese Medicine for Prevention and Cure of Metabolic Diseases, Chongqing 400016, China; ^3^Chongqing College of Traditional Chinese Medicine, Chongqing 402760, China; ^4^Department of Chinese Traditional Medicine, The First Affiliated Hospital of Chongqing Medical University, Chongqing 400042, China

## Abstract

**Background:**

The composition and metabolic activities of gut microbiota are strongly interconnected with cardiac fibrosis (CF) and heart failure (HF). Qige Huxin formula (QHF), a traditional Chinese medicine (TCM) formulation originating from a classical Fangji Huangqi decoction, has been widely used to clinically treat HF for decades. However, it is still unclear whether QHF alleviates CF by modulating gut microbiota and intestinal integrity.

**Purpose:**

This study aimed to investigate the cardioprotective effects of QHF in isoprenaline-induced CF through modulating gut microbiota and intestinal integrity.

**Methods:**

Fifty mice were randomly divided into five groups after one week of acclimatization feeding: control group, model group, 2.56 g/kg/d group (low-dose QHF), 5.12 g/kg/d group (high-dose QHF), and meto group (15 mg/kg/d). The CF model was established by subcutaneously injecting the mice with isoprenaline (10 mg/kg/d for 14 days), followed by QHF treatment. The heart volume, cardiac weight index (CWI), serum myocardial enzymes, serum inflammatory cytokines, serum lipopolysaccharide, histopathology of the heart and colon tissues, ZO-1, and occludin of colon tissues were then measured. Fecal samples from mice were analyzed using 16S rRNA sequencing.

**Results:**

QHF treatment significantly reduced heart volume, CWI, and serum CK and CK-MB levels, attenuated cardiac histopathological alterations, and alleviated CF. QHF treatment also downregulated TNF-*α*, IL-1*β*, and IL-6 in serum. Moreover, QHF treatment decreased the serum level of lipopolysaccharide and maintained intestinal integrity by upregulating ZO-1 and occludin. The 16S rRNA microbiota analysis revealed that QHF treatment increased the relative abundance of *Marvinbryantia* and *Phascolarctobacterium*.

**Conclusions:**

QHF treatment exerts cardioprotective effects through modulating gut microbiota, protecting intestinal integrity, and alleviating inflammation. This study shows that gut microbiota may enhance heart-gut interaction.

## 1. Introduction

Cardiac fibrosis (CF) is characterized by fibroblast activation, fibroblast transformation into myofibroblast, and the deposition of the extracellular matrix (ECM) [1]. CF is an inevitable pathological process and an irreversible contributing factor of heart failure (HF) [2, 3]. HF has the characteristics of high morbidity, mortality, and dismal prognosis, and seriously affects the health and life of patients [4]. However, the relevant mechanisms of CF remain unclear. There are no effective treatments to halt or reverse the progression of fibrosis since the current medical therapies for CF are very limited. Therefore, there is an urgent need to discover novel mechanisms of CF and identity new therapeutic targets for effective antifibrosis and delaying the progression of CF to HF.

Many studies have indicated that the composition and metabolic activities of gut microbiota are intimately interconnected with CF and HF [5–7]. HF decreases cardiac output, resulting in intestinal wall ischemia, intestinal mucosal edema, and destruction of intestinal integrity, eventually increasing intestinal permeability (“leaky gut”) [8]. Previous studies have also indicated that gut microflora dysbiosis may damage intestinal integrity, increase intestinal mucosal permeability, and lead to the leakage of inflammatory metabolites into the blood, thus triggering an inflammatory response that results in CF and HF [9–11]. Therefore, modulating gut microflora and protecting intestinal integrity can ameliorate CF and delay the progression of CF to HF.

Qige Huxin formula (QHF), a traditional Chinese medicine (TCM) formulation originating from a classical Fangji Huangqi decoction, has been widely used to clinically treat HF for decades. Pharmacological and phytochemical evidence has demonstrated that QHF contains various bioactive components and has multiple pharmacological functions, including antioxidation [12], antifibrosis [13], anti-inflammation [14], and anticardiac remodeling [15] through the synergic effects of multiple active ingredients. Recent studies have also indicated that astragaloside IV and puerarin, the main effective ingredients of QHF, can modulate gut microbiota and promote intestinal mucosal repair [16, 17]. These studies indicate that QHF can attenuate CF by modulating gut microbiota and protecting intestinal integrity. This study aimed to investigate whether QHF can ameliorate CF by modulating gut microbiota, intestinal integrity, and inflammation.

## 2. Materials and Methods

### 2.1. Animals and Drugs

Fifty specific pathogen-free (SPF) male Kunming mice (weight, 22–26 g) were obtained from the Laboratory Animal Center of Chongqing Medical University (CQMU, Chongqing, China). The mice were then acclimated to the environment for one week. An air-conditioned environment with standardized conditions (relative humidity: 40%–60%, ambient temperature: 20°C–22°C, and light: 12 h light/dark cycle) was provided for the mice during the experiment. Ad libitum food and water were provided to each mouse during the experiments. Animal experiments were conducted following the guidelines for care and use of laboratory animals established by the International Council on Research Animal Care and the Chinese Animal Welfare Committee. Experimental animal protocols were approved by the Animal Experiments Ethical Review Committee of CQMU. QHF consists of *Astragali Radix (Huangqi*, 6 g*), Puerariae Lobatae Radix (Gegen,* 3 g*), Atractylodis Macrocephalae Rhizoma (Baizhu,* 4.5 g*), Curcumae Longae Rhizoma (Jianghuang,* 2.7 g*), Stephaniae Tetrandrae Radix (Fangji,* 0.72 g), and *Fritillariae Thunbergii Bulbus (Zhebeimu,* 3 g). Animal equivalent doses were converted from human doses based on body surface area to calculate the oral doses of drugs given to the mice [18]. The lavage doses of the low-dose and high-dose QHF groups were 2.56 g/kg/d and 5.12 g/kg/d, respectively. QHF was purchased from Guangdong Yifang Pharmaceutical Co., Ltd. (Guangdong, China) and identified by Professor Wenfu Cao. Metoprolol tartrate tablets (product batch number: 2012A48) were sourced from AstraZeneca Pharmaceuticals (China) Co., Ltd. (Taizhou, China).

### 2.2. CF Model Establishment and Grouping

**Figure 1:**
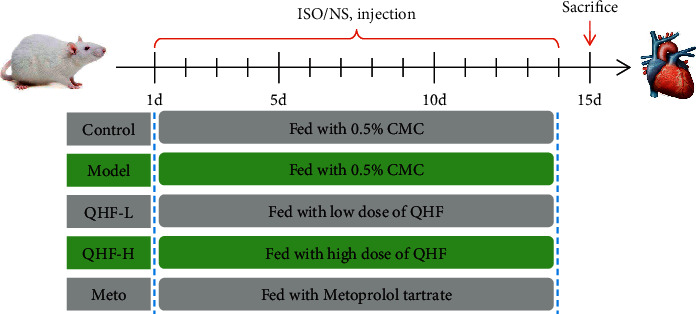
The experimental timeline and schematic design of the study.

The mice were randomly divided into five groups (*n* = 10 per group) after one week of acclimatization feeding. The CF model was established as previously described [3, 19]. Mice in the control group were subcutaneously administered 0.9% sterile saline solution at the back of the neck. Mice in other groups were subcutaneously injected with isoproterenol (ISO, 10 mg/kg/d) for 14 days. Mice in the control and model groups were orally fed 0.5% carboxymethyl cellulose (CMC) for 14 days. Mice in the meto group were orally fed metoprolol tartrate (15 mg/kg/d) for 14 days, whereas mice in the low-dose QHF (QHF-L) and the high-dose QHF (QHF-H) groups were treated with QHF doses of 2.56 g/kg/d and 5.12 g/kg/d for 14 days, respectively. All experimental drugs were dissolved in a 0.5% aqueous CMC solution.  Figure 1 illustrates the experimental timeline and schematic design of the study.

### 2.3. Plasma Biomarkers and Cardiac Weight Index

The serum levels of myocardial enzymes, including creatine kinase (CK) and creatine kinase-MB (CK-MB), were measured using a Rayto Chemray 800 automatic biochemical analyzer. The serum level of lipopolysaccharide (LPS) was detected using an endotoxin detection kit (Nanjing Jiancheng Bioengineering Research Institute; Nanjing, China). Blood was collected, and then, the mice were euthanized by inhalation of 5% isoflurane. The mice's chests were opened and the hearts were fully exposed. The heart tissues were immediately removed and weighed. The Cardiac Weight Index (CWI) was calculated to evaluate cardiac function. CWI (mg/g) = heart weight (HW, mg)/body weight (BW, g) × 100%.

### 2.4. Histopathology Assay and Quantification of Cardiomyocyte Sizes

The hearts and colons of freshly excised mice were fixed in 4% paraformaldehyde at room temperature for 24 hours. The tissues were embedded in paraffin and cut into 5 *μ*m sections using a microtome (Leica) after fixation. The heart tissue sections were dewaxed and stained with HE and Masson. The HE staining kit (Servicebio, G1003) and the Masson staining kit (Servicebio, G1006) were purchased from Wuhan Servicebio Technology Co., Ltd (Wuhan, China). An Olympus BX53 biological microscope (Olympus, Tokyo, Japan) was used to photograph at least five positive areas randomly selected from each section. The area of positive staining was quantified using Image-Pro Plus 6.0 (Media Cybernetics, Bethesda, MD, USA). The myocyte cross-sectional areas in HE-stained vertical sections were measured (*n* = 50 cells per animal) using Image-Pro Plus 6.0 to determine cardiac hypertrophy [20].

### 2.5. Measurement of Inflammatory Cytokines

The serum levels of TNF-*α*, IL-1*β*, and IL-6 were analyzed using commercial kits following the manufacturer's instructions. These commercial kits were purchased from Quanzhou Jiubang Biotechnology Co., Ltd. (Quanzhou, China).

### 2.6. Immunohistochemical Staining

Immunohistochemistry staining of *α*-smooth muscle actin (*α*-SMA), zonula occludens-1 (ZO-1), and occludin were carried out following the standard protocol. Paraffin-embedded sections (5 *μ*m) were dewaxed in xylene, and then rehydrated with gradient ethanol, followed by antigen retrieval in a microwave for 15 min using sodium citrate buffer (Servicebio, G1201, pH 6.0). The slices were protected from light and incubated in 3% H_2_O_2_ (Servicebio, G0115) at ambient temperature for 25 minutes to deplete endogenous peroxidase. The sections were blocked with 3% bovine serum albumin (Servicebio, G5001) for 30 minutes, and then washed thrice using phosphate-buffered saline (PBS, PH 7.2) (five minutes each) at room temperature. Afterward, the sections were incubated with antibodies specific for *α*-SMA (Servicebio, GB111364, 1 : 1000), ZO-1 (Servicebio, GB111981, 1 : 400), and occludin (Servicebio, GB111401, 1 : 600) at 4°C overnight. The sections were washed thrice (five minutes each) using PBS (pH 7.4) after incubation with the primary antibody. The sections were then incubated with the appropriate secondary antibody at room temperature for 50 minutes. The sections were washed again thrice (5 minutes each) as mentioned above and stained with a freshly prepared DAB reagent (Servicebio, G1211). The sections were counterstained with hematoxylin (Servicebio, G1004), dehydrated in alcohol gradients and xylene, and then blocked with neutral gum. Five positive areas of each section were randomly selected under a light microscope (Olympus BX53) at a magnification of x400. The positive staining area, defined as brown-yellow, was quantified using Image-Pro Plus 6.0.

### 2.7. 16S rRNA Sequencing

16S rRNA sequencing was performed following the manufacturer's instructions. The DNA of the microorganisms was extracted from fecal samples using an E.Z.N.A. soil DNA kit (Omega Bio-Tek, Norcross, GA, U.S.). The DNA purity was confirmed using 1% agarose gel electrophoresis. Quality checks were performed on all DNA samples and concentration was determined using NanoDrop 2000 spectrophotometers (Thermo Fisher Scientific, Wilmington, DE, USA). The variable regions V_3_-V_4_ of the 16S rRNA gene were amplified using the following primers: 338F (5′-ACTCCTACGGGAGGCAGCAG-3′) and 806R (5′-GGACTACHVGGGTWTCTAAT-3′). The PCR reaction conditions were as follows: 95°C for 30 s, 55°C for 30 s, and 27 cycles at 72°C for 45 s. Each PCR mixture (20 *μ*L) contained 4 *μ*L TransStart FastPfu buffer (5 ×), 0.8 *μ*L of each primer (5 *μ*M), 0.4 *μ*L TransStart FastPfu DNA polymerase, 2 *μ*L of 2.5 mM deoxynucleoside triphosphates, and 10 ng of extracted DNA, and ddH_2_O was added to make up 20 *μ*L. Purification of DNA fragments was conducted using an AxyPrep DNA gel extraction kit (Axygen Biosciences, Union City, CA, USA). DNA quantification was carried out using a Quantus™ fluorometer (Promega, Madison, WI, USA). Finally, sequencing libraries were prepared using a NEXTflex™ rapid DNA-seq kit (Bioo Scientific, USA), and then, the sequencing was conducted on the Illumina MiSeq PE300 platform (Illumina, San Diego, CA).

The raw image data files were converted into the original sequenced reads after base calling analysis. The final results were transformed into the FASTQ (abbreviated as fq) format using Bcl2Fastq [21]. FASTQ paired-end raw data were merged with FLASH [22] software. The low-quality (score <20) and the short (<50 bases) sequences were removed after the screening of raw sequences. The remaining qualifying sequences were then spliced. The UCLUST algorithm in QIIME2 [23] software was used to cluster and annotate the operational taxonomic units at a similarity threshold of 97%. Alpha and beta diversity analyses were conducted according to the clustering results. The naive classifier in QIIME2 was used for species taxonomic analysis of ASVs based on the SILVA 16S rRNA database. The sequencing data underwent a diversity analysis using the free online platform of Majorbio Cloud (https://www.cloud.majorbio.com).

### 2.8. Statistical Analysis

Data were expressed as mean ± standard deviation (SD) and analyzed with SPSS 26.0 statistical software (SPSS Inc., Chicago, USA). One-way analysis of variance (ANOVA) was used to compare variance among multiple groups. All graphs were plotted in GraphPad Prism 9.20 (GraphPad Software, California, USA). *P* < 0.05 was considered statistically significant.

## 3. Results

### 3.1. QHF Reduced CWI and Regulated Body Weight

**Figure 2:**
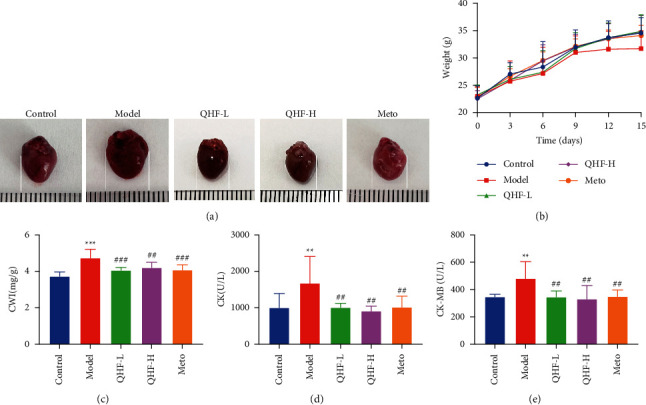
QHF regulated body weight and deceased myocardial enzymes in mice with CF. (a) Cardiac morphology. (b) Body weight in mice. (c) Cardiac Weight Index. (d, e) Levels of CK and CK-MB in serum (*n* = 6). Data are presented as mean ± SD. ^*∗∗*^*P* < 0.01 and ^*∗∗∗*^*P* < 0.001 versus the control group; ^##^*P* < 0.01^###^*P* < 0.001 versus the model group.

Cardiac morphology and CWI were observed and measured to determine how QHF protects against ISO-induced myocardial damage. The model group had a significantly larger heart volume than the control group. In contrast, the QHF group had a reduced heart volume compared with the model group (Figure 2(a)). Additionally, CWI significantly differed between the model group and other groups after 14 days (Figure 2(c)). Moreover, the body weight of each mouse was measured and recorded every three days throughout the experiment. Mice in the five groups had similar body weights at the beginning of the experiment (Figure 2(b)). However, the body weight in the model group showed a downward trend at the end of the experiment, while it showed an increasing trend in the other groups (Figure 2(b)). Nevertheless, the body weight was not significantly different among the experimental groups.

### 3.2. QHF Improved ISO-Induced Myocardial Damage

The serum levels of myocardial injury markers CK and CK-MB were detected. The serum levels of CK and CK-MB significantly increased in the model group and obviously deceased in the QHF groups (Figures 2(d), 2(e)), indicating that QHF can protect against ISO-induced myocardial damage.

### 3.3. QHF Ameliorated ISO-Induced Histopathological Changes

**Figure 3:**
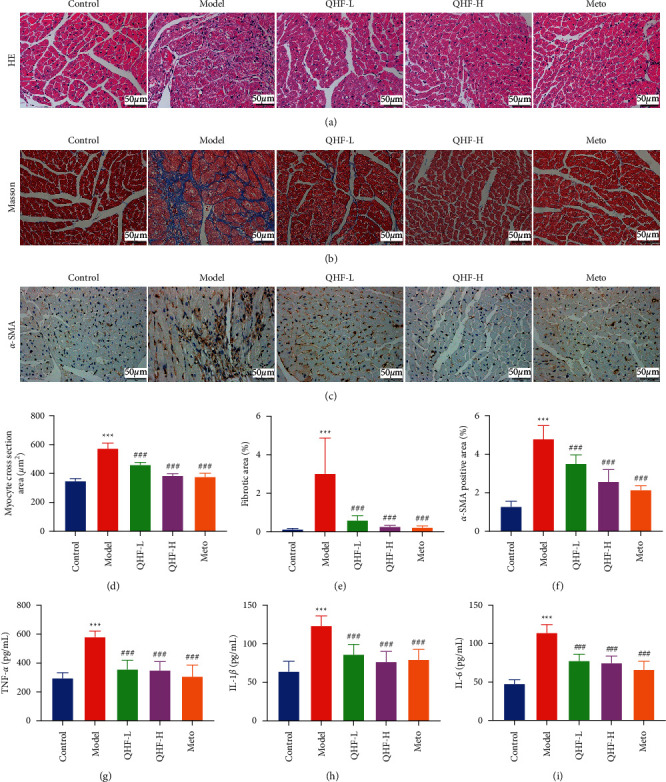
QHF alleviated cardiac fibrosis and decreased inflammation in mice with CF. ((a, b, c) × 400) Representative images of cardiac fibrosis reflected by HE, Masson, and immunohistochemical staining. (d) The myocyte cross section area. (e, f) Quantification of interstitial fibrosis and *α*-SMA (*n* = 6). (g, h) (i) Levels of TNF-*α*, IL-1*β*, and IL-6 in serum (*n* = 8). Data are presented as mean ± SD. ^*∗∗∗*^*P* < 0.001 versus the control group; ^###^*P* < 0.001 versus the model group.

Myocardial histopathological changes, stained with hematoxylin-eosin (HE), were observed using a light microscope. Myocardial fibers in the model group were severely damaged, with focal necrosis, myocardial structural disorder, and inflammatory cell infiltration (Figure 3(a)). However, these histopathological changes were remarkably reduced by QHF treatment. The myocyte cross-sectional area is a measure of cardiac hypertrophy, which is closely related to CF [24]. Herein, the myocyte cross-sectional area was significantly elevated in the model group and was attenuated following QHF treatment (Figure 3(d)). These results indicated that QHF can prevent ISO-induced CF.

### 3.4. QHF Alleviated ISO-Induced Cardiac Fibrosis

Cardiac fibrosis resulting from myocardial interstitial collagen deposition among the ECM can be investigated by Masson staining. The area of collagen deposits in the interstitial of the myocardium was significantly different between the model and control groups (Figures 3(b), 3(e)). Compared with the model group, QHF significantly reduced the area of collagen deposition in ECM. Cardiac fibroblasts differentiate into myofibroblasts, which secrete large amounts of collagens and express abundant *α*-SMA, thus promoting CF [25]. We further examined the expression level of *α*-SMA in myocardial tissue by immunohistochemistry. The expression level of *α*-SMA was significantly upregulated in the model group, while it was markedly reduced by QHF treatment (Figures 3(c), 3(f)). These results demonstrated that QHF can improve collagen deposition and attenuate ISO-induced CF.

### 3.5. QHF Decreased ISO-Induced Inflammation

Inflammation is a protective response to various injuries; however, excessive inflammation may damage the failing heart, which contributes to CF and cardiac dysfunction [26]. The serum levels of proinflammatory cytokines, including TNF-*α*, IL-1*β*, and IL-6 were higher in the model group than those in the control group (Figures 3(g)–3(i)). However, QHF treatment obviously decreased the levels of these inflammatory cytokines, indicating that QHF can decrease ISO-induced inflammation.

### 3.6. QHF Protected ISO-Induced Intestinal Integrity

**Figure 4:**
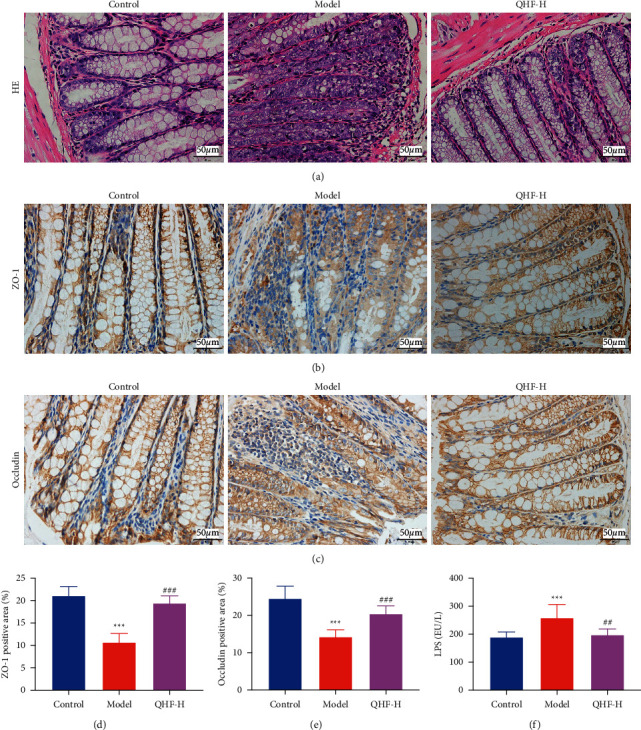
QHF protected ISO-induced intestinal integrity in mice with CF. ((a) × 400) The HE staining of colon tissues. ((b), (c) × 400) Representative images of immunohistochemical staining for ZO-1 and occludin in intestinal mucosa. (d, e) Quantification of ZO-1 and occludin (*n* = 6). (f) Levels of LPS in serum (*n* = 6). Data are presented as mean ± SD. ^*∗∗∗*^*P* < 0.001 versus the control group; ^##^*P* < 0.01 and ^###^*P* < 0.001 versus the model group.

The intestinal barrier can effectively prevent harmful substances, such as bacteria and toxins from passing through intestinal mucosa into the body's tissues, organs, and blood circulation [10]. Therefore, maintaining intestinal integrity is essential for cardiac health. HE staining revealed destruction of goblet cells and inflammatory infiltration of the colon in the model group (Figure 4(a)). However, QHF treatment (high dose) effectively repaired intestinal damage and reduced severe histologic inflammation. Tight junction proteins (TJPs), including ZO-1 and occludin, are the major elements in modulating the permeability in the intestine and maintaining intestinal integrity [27]. In this study, ZO-1 and occludin proteins were significantly downregulated in the colon tissues of the model group compared with those of the control group. However, QHF treatment (high dose) markedly upregulated ZO-1 and occludin (Figures 4(b)–4(e)).

Lipopolysaccharide (LPS) is a sensitive biomarker of barrier strength and intestinal permeability [28, 29]. LPS crosses the intestinal barrier into the bloodstream after intestinal damage and disperses throughout the body. Therefore, the serum level of LPS can indirectly reflect intestinal integrity. In this study, the serum level of LPS was significantly upregulated in the model group compared with the control group (Figure 4(f)). However, QHF treatment (high dose) decreased the serum level of LPS, demonstrating that QHF can reduce ISO-induced intestinal permeability and protect intestinal integrity.

### 3.7. QHF Improved ISO-Induced Gut Microbial Dysbiosis

**Figure 5:**
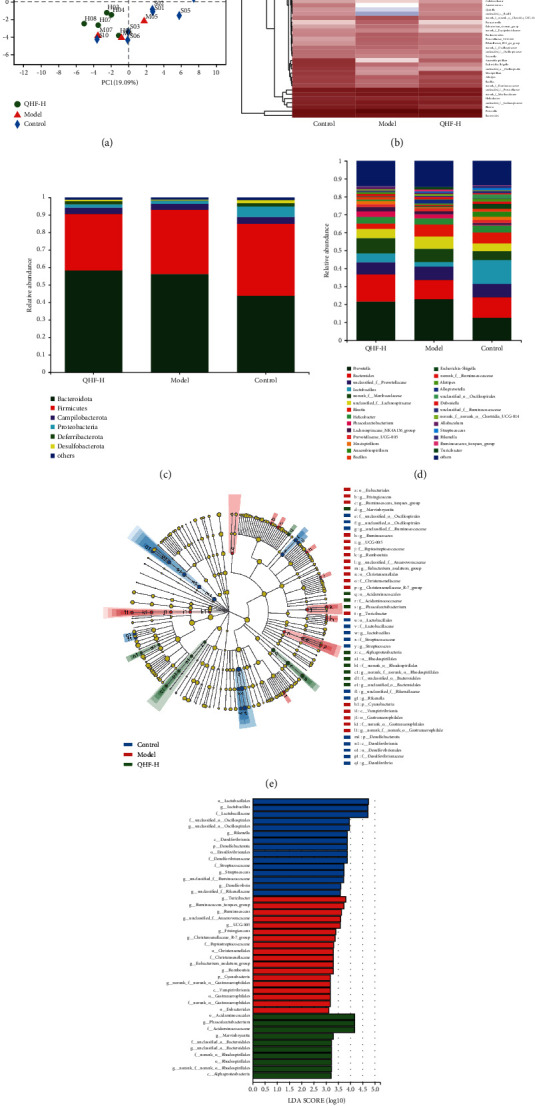
QHF improved ISO-induced gut microbial dysbiosis in mice with CF. (a) PCA. (b) Heatmap analyses of the dominant genus. (c, d) Relative abundance of intestinal microbes at the phylum and genus levels, respectively. (e) Taxonomy cladogram. (f) Gut microbial taxa (LDA threshold of 4.0).

16S rRNA sequencing was used to assess whether QHF treatment (high dose) can regulate gut microbiota composition in mice. The principal components analysis (PCA) was performed to assess the composition of gut microbiota. The gut microbiota compositions of the three groups were distinctly separated, indicating that they had significantly different gut microbiota compositions (Figure 5(a)). The gut microbial community structure changes were analyzed using heatmap analysis. Compared with the control group, the gut microbiota of the model group was significantly different, whereas QHF treatment (high dose) reversed the microbe changes in ISO-induced mice (Figure 5(b)). The phylum-level community structure composition histogram revealed that the structure and abundance of the fecal microbial community were altered. *Bacteroidetes* and *Firmicutes* were the most dominant phyla (over 85%) in all groups (Figure 5(c)). At the genus level, *Prevotella* had the highest relative abundance, followed by *Bacteroides* (Figure 5(d)). Compared with the model group, QHF treatment (high dose) increased the relative abundance of *Lactobacillus* and *Bacteroides*, while it decreased the relative abundance of *Escherichia-Shigella* (Figure 5(d)). The differential bacteria composition was identified using the LEfSe and taxonomy cladogram. QHF treatment (high dose) obviously increased the relative abundance of family *Marvinbryantia* and *Phascolarctobacterium* at the genus levels (Figures 5(e), 5(f)). These results demonstrated that QHF can modulate gut microbiota composition, thus alleviating CF.

## 4. Discussions

Cardiac fibrosis (CF) is a continuous and dynamic pathologic process characterized by transforming resident fibroblasts into myofibroblasts and excess deposition of collagens in the myocardium [30]. The pathophysiology of CF can be reversed at the early stage [31]. However, it can lead to continuous accumulation of ECM if left untreated, eventually resulting in cardiac dysfunction and heart failure [32]. Nevertheless, the mechanisms of CF are unknown, and there is no efficacious treatment to halt the progression of CF [33]. A significant amount of research has focused on the therapeutic potential of TCM in the prevention and treatment of CF [34]. This study investigated whether QHF can achieve the antifibrosis effects in ISO-induced mice by regulating gut microbiota, intestinal integrity, and inflammation.

Our data indicated that QHF treatment could significantly alleviate ISO-induced myocardial injury and cardiac fibrosis by reducing serum levels of CK and CK-MB, heart volume, CWI, and cross-sectional area of cardiomyocytes. Moreover, HE staining demonstrated that QHF treatment corrected the structural disorder of cardiomyocytes and decreased inflammatory cell infiltration and myocardial necrosis. Masson staining displayed that QHF treatment inhibited myocardial collagen deposition. Immunohistochemistry staining also showed that QHF treatment markedly downregulated *α*-SMA in myocardial tissue.

Previous studies have indicated that inflammatory cytokines are significantly upregulated in myocardial fibrosis [35]. Inflammatory cytokines can promote the transformation of fibroblasts into myofibroblasts by increasing the expression of proteins involved in ECM, thus accelerating CF progression [26]. An inflammatory response is immediately triggered when the body or cells are stimulated, thus making inflammatory cells immediately release various proinflammatory mediators, including TNF-*α*, IL-1*β*, and IL-6. These substances, as proinflammatory mediators, stimulate the proliferation and activation of the resident fibroblasts and their transformation into myofibroblasts [36], thus promoting the deposition of matrix proteins in the ECM. Previous studies have exhibited that decreasing proinflammatory factors can ameliorate myocardial fibrosis [37–39]. In our present study, the serum levels of proinflammatory cytokines, including TNF-*α*, IL-1*β*, and IL-6, were increased significantly in the model group. However, QHF treatment decreased the serum levels of inflammatory cytokines, illustrating that QHF treatment can protect against ISO-induced CF by inhibiting inflammation.

The intestinal mucosal barrier consists of TJPs and intestinal epithelial cells. It is the first physiological barrier for the human body to resist harmful intestinal microorganisms and their metabolites. Among the most important TJPs, ZO-1 and occludin play a crucial role in regulating intestinal permeability [40]. Bacteria and their metabolites, especially LPS, enter blood through intestinal mucosa when intestinal permeability increases, leading to bacterial infection and inflammatory response, thus inducing heart injury [41]. In this study, QHF treatment upregulated ZO-1 and occludin in colonic mucosa, and suppressed the serum LPS level. Furthermore, HE staining showed that QHF treatment increased the number of goblet cells in intestinal mucosa, reduced inflammatory cell infiltration, and effectively repaired the intestinal injury. These findings showed that QHF exerted a protective effect on intestinal integrity.

A lot of evidence has demonstrated that the imbalance of intestinal flora promotes CF progression [42, 43]. Herein, QHF treatment regulated the gut microbiota structure. QHF consumption significantly increased the relative abundance of *Bacteroides* and *Mucispirillum* in mice feces, while it decreased the relative abundance of *Escherichia-Shigella*. Several studies have shown that microbiota with low *Bacteroides* abundance or high *Helicobacter* abundance can increase the risk of cardiovascular diseases [44, 45]. In this study, QHF treatment significantly increased the relative abundances of *Marvinbryantia* and *Phascolarctobacterium* at the genus level. *Marvinbryantia*, a genus of *Firmicutes*, can produce short-chain fatty acids (SCFAs) [46]. SCFAs are mainly produced by the fermentation of dietary fiber. SCFA reduction increases the risk of CF and HF [47]. *Marvinbryantia* is a beneficial intestinal bacterium that maintains the diversity and function of gut microbiota and can improve human health. For instance, it can protect the intestinal epithelium cells from destruction, and has anti-inflammatory properties [48]. Furthermore, QHF treatment improved the relative abundance of *Phascolarctobacterium*. A recent study found that *Phascolarctobacterium* is negatively correlated with the IL-6 level [49]. Therefore, *Marvinbryantia* and *Phascolarctobacterium* promote the antifibrosis effects of QHF treatment in ISO-induced mice.

## 5. Conclusion

Collectively, QHF treatment exerts obvious cardioprotective effects in ISO-induced cardiac fibrosis mice by modulating gut microbiota, protecting intestinal integrity, and inhibiting inflammation. This study provides a new therapeutic strategy for QHF to prevent and treat cardiac fibrosis.

## Data Availability

All datasets used and/or generated during the current study are available from the corresponding author upon request.

## Abbreviations

ANOVA: One-way analysis of variance
BW: Body weight
CF: Cardiac fibrosis
CK: Creatine kinase
CK-MB: Creatine kinase-MB
CMC: Carboxymethyl cellulose
CQMU: Chongqing medical university
CWI: Cardiac weight index
ECM: Extracellular matrix
HE: Hematoxylin-eosin
HF: Heart failure
HW: Heart weight
ISO: Isoprenaline
LPS: Lipopolysaccharide
Meto: Metoprolol tartrate tablet
PCA: Principal components analysis
QHF: Qige huxin formula
SCFAs: Short-chain fatty acids
SD: Standard deviation
SPF: Specific pathogen-free
TCM: Traditional Chinese medicine
TJPs: Tight junction proteins
ZO-1: Zonula occludens-1
*α*-SMA: *α*-Smooth muscle actin.
